# Photo-Piezojunction Coupling Effect in n-3C-SiC/p-Si Heterojunction – A Platform for Self-Powered Strain-Sensing Applications

**DOI:** 10.1021/acsami.5c02290

**Published:** 2025-04-17

**Authors:** D. H. Dang Tran, Tuan-Hung Nguyen, Cong Thanh Nguyen, Erik W. Streed, Nam-Trung Nguyen, Van Thanh Dau, Dzung Viet Dao

**Affiliations:** † Queensland Micro- and Nanotechnology Centre, Griffith University, 170 Kessels Road, Brisbane, Queensland 4111, Australia; ‡ Institute for Glycomics and Centre for Quantum Dynamics, Griffith University, Parklands Drive, Gold Coast, Queensland 4222, Australia; § School of Engineering and Built Environment, Griffith University, Parklands Drive, Gold Coast, Queensland 4222, Australia

**Keywords:** n-3C-SiC/p-Si heterojunction, photon energy harvesting, multifunctional sensors, photovoltaic effect, photo-piezojunction coupling effect

## Abstract

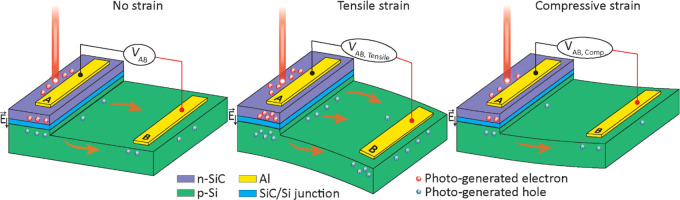

It is beneficial to investigate multifunctional self-powered sensors with high sensitivity and energy-scavenging capabilities, which are essential for the development of a smart infrastructure in the era of 5G and Internet of Things (IoT). This paper reports the photo-piezojunction coupling effect, i.e., the coupling of piezojunction effect with photovoltaic effect, in an n-type 3C-SiC/p-type Si heterojunction (n-3C-SiC/p-Si) and demonstrates it through a proof-of-concept self-powered strain-sensing device. The device exhibits superior photon energy-harvesting capability, generating 24.54 mV with a laser power of only 10 μW, approximately five times higher than that of reported devices utilizing the lateral photovoltaic effect, and an exceptionally high strain sensitivity, achieving |(Δ*V*/*V*)/ε| ratios of 43.14 and 21.34 for tensile and compressive strain, respectively, which are 4.3 times more than that of devices reported in previous studies. The findings of this study significantly advance the understanding of the photo-piezojunction coupling effect in n-3C-SiC/p-Si heterostructures, laying the foundation for the development of multifunctional sensor systems capable of harvesting photon energy.

## 1 Introduction

The rapid development of smart infrastructure requires monitoring systems capable of continuously and reliably feeding back environmental signals, allowing accurate predictions and early prevention of potential issues, which ultimately contributes to safer and more convenient living standards.^1^ However, maintaining a constant power supply and limited battery life remain the challenges for conventional wired systems.^2^ A potential solution to this issue involves investigating new methods to integrate energy-scavenging capabilities with sensing functions into a single sensor package.^3,4^ This approach allows the fabrication of multifunctional sensors, e.g., strain sensors^5,6^ or accelerometers,^7^ capable of monitoring the external environmental signals while simultaneously harvesting the available energy sources from the environment.

Among the energy-harvesting methods, the photovoltaic effect can effectively collect solar photon energy, which is one of the most readily available energy sources. Since its first observation in the silicon p–n junction in 1959,^8^ the photovoltaic effect in various semiconductor materials and semiconductor heterojunctions has been intensively investigated^9−13^ due to its high energy conversion efficiency and low-cost mass producibility. According to recent studies, the coupling of photovoltaic effect with other physical effects can enhance the performance of each individual effect.^14^ One example is the coupling of photovoltaic effect with strain-induced piezoelectric polarization, known as piezo-phototronic coupling effect, which can enhance the optical performance in ZnO and GaN materials.^15^ Another method involves applying an atomic force over a nanoscale contact area to modify the local polarization, known as flexo-photovoltaic effect, which can activate the local photovoltage effect in the confined strained area, enabling photon energy harvesting in that nanoscale region.^16−18^ Besides coupling with the piezoelectric effect as mentioned, the photovoltaic effect can also combine with the piezoresistive effect, known as piezo-optoelectronic coupling effect, to boost the mechanical strain sensitivity in strain sensors.^19,20^ Interestingly, recent research demonstrates a large piezoresistive effect across the junction region, known as piezojunction effect, in heterojunction material.^21^ Combining the piezojunction effect with photovoltaic effect results in the photo-piezojunction effect, which can further boost the mechanical strain sensitivity through additional photogenerated charges, allowing the device to sense mechanical strain and harvest photon energy simultaneously.

In this paper, we report the photo-piezojunction coupling effect in a heterostructure device made from an n-type 3C-SiC thin film grown on p-type Si substrate, forming an n-3C-SiC/p-Si heterojunction, and demonstrate the effect with a proof-of-concept strain-sensing device capable of simultaneously harvesting photon energy. The sensor device generates 25.54 mV under the illumination of a red laser with a power of only 10 μW, i.e., almost five times higher than that of devices of similar materials utilizing the lateral photovoltage effect.^22^ Furthermore, the device features a top-side design with electrodes positioned on the top surfaces of both SiC and Si layers, allowing flexibility in strain direction selection compared to the top-bottom electrodes design,^21^ where the strain direction is limited to only vertical, resulting in complex strain patterns. The strain sensor device in this study demonstrates excellent mechanical strain-sensing performance, outputting an absolute strain sensitivity ratio |(Δ*V*/*V*)/ε| of 43.14 and 21.34 for tensile and compressive strain, respectively, 4.3 times more sensitive than that of devices with similar structures^23^ and ten times higher than that of conventional metal strain gauges, which typically have a gauge factor of only around 2.^24^ The underlying mechanisms are explained based on the theories of photovoltaic effect, energy valleys warping and shifting, and charge carrier transfer in both the conduction band of n-3C-SiC and the valence band of p-Si. The findings of the research provide a fundamental theory background for future design of sensing devices utilizing the photo-piezojunction effect.

## 2 Concept

**Figure 1:**
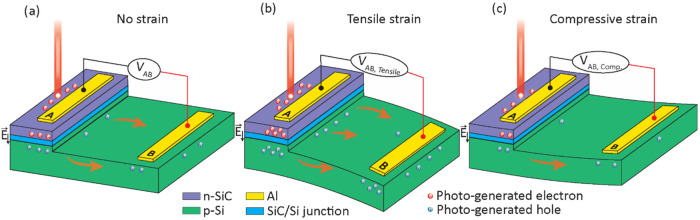
Concept of photo-piezojunction coupling effect in a light-harvesting strain sensor under red laser illumination in the cases (a) no strain, (b) tensile strain, and (c) compressive strain.

Figure 1 illustrates the concept of the photo-piezojunction coupling effect in a light-harvesting strain-sensing device consisting of two layers, an n-doped SiC (n-SiC) layer grown on a p-doped Si (p-Si) substrate, and aluminum electrodes are deposited on each layer to collect the charge carriers within the SiC layer and Si substrate. The SiC and Si layers have different doping types, resulting in different charge carrier concentrations in each layer; specifically, n-SiC layer has more electrons, while p-Si substrate has more holes, causing the charge carriers to move across the interface between the layers and combine with the charge carriers of opposite type in the other layer.

This process eventually reaches equilibrium, creating the n-SiC/p-Si junction, a region free of moving charges with an electric field *E⃗* pointing downward to the p-Si substrate. Illuminating the device with a red laser generates electron–hole pairs (EHPs), which are subsequently separated by electric field *E⃗* across the junction. The electric field *E⃗* sweeps the electrons upward to the n-SiC layer and holes downward to the p-Si substrate, resulting in a voltage *V*_AB_ measured between two electrodes A and B (Figure 1a). Application of external force to the device activates the sensor’s strain-sensing property, causing the output voltage *V*_AB_ to vary. The trend of *V*_AB_ reflects the direction and magnitude of the strain exerted on the sensing element. Specifically, under tensile strain, the device outputs a larger voltage *V*_AB,tensile_ as more charge carriers are populated at the junction area (Figure 1b), while under compressive strain, the device outputs a smaller voltage *V*_AB,compressive_ due to a decrease in charge carriers population (Figure 1c).

## 3 Fabrication and Experimental Setup

We have designed and fabricated a proof-of-concept device for characterizing the photo-piezojunction coupling effect. The fabrication process follows 11 steps as illustrated in Supporting Information Figure S1. The process started with a commercialized p-type Si wafer with a thickness of 380 μm, boron doped, and a doping concentration of 7 × 10^14^ atom/cm^3^. The Si wafer was cleaned following the standard Radio Corporation of America (RCA) procedure to eliminate the dust and organic substances from the surface (Step 1). Subsequently, a single-crystalline unintentional n-type doped SiC thin layer with a thickness of 500 nm was epitaxially grown on the Si wafer using a low-pressure chemical vapor deposition process (LPCVD) at 1000 °C, employing two precursors: propylene (C_3_H_6_) and silane (SiH_4_).^25^ The SiC layer thickness of 500 nm was measured using Nanometrics Nanospec 210 instruments, and the doping concentration of the n-SiC layer was approximately 3 × 10^17^ atoms/cm^3^, determined using the hot probe technique^26^ (step 2). The diode structure was constructed by etching portions of the SiC layer into diode patterns utilizing the photolithography techniques combined with deep reactive ion etching (DRIE) method, exposing the surfaces of both SiC and the Si layer facing upward (step 3–4). To ensure an ohmic contact between the subsequent layer, aluminum (Al) electrodes layer, and the diode surfaces, the excess photoresist on the diode surfaces was thoroughly cleaned using Tegal 915, preparing the surfaces for direct deposition of aluminum electrodes on the top. In the next step, an aluminum layer 500 nm thick was sputtered on the diode surfaces, followed by a photoresist layer deposited on the top using the spin coating method (step 6–7). The electrodes design was transferred to the photoresist layer using the photolithography method, specifically, exposing the design under ultraviolet (UV) light using the maskless aligner MLA150 (Heidelberg Instruments) and submerging the wafer into the developer solution, developing the electrode pattern exposed to the UV light while dissolving the remaining photoresist area without UV light exposure (step 8). Subsequently, the Al area without the cover of photoresist mask was removed using the wet etching method, resulting in aluminum electrodes in two areas, one positioned on the SiC layer and the other on the Si side of the diode structure (step 9). The electrodes were aligned in ⟨100⟩ direction, where the piezoresistive effect of horizontal strain is minimal,^27^ allowing the characterization to focus only on the strain effect of the vertical direction, i.e., [001] direction of the n-SiC/p-Si heterojunction. The electrodes were spaced 500 μm apart from each other, with dimensions of 200 μm in width and 500 μm in length. Finally, the wafer was diced into individual beam devices measuring 20 mm in length and 5 mm in width (step 10). The wire bonding technique was used to connect the Al electrodes on the device to an external printed circuit board (PCB), improving the handleability during characterization experiments (step 11).

**Figure 2:**
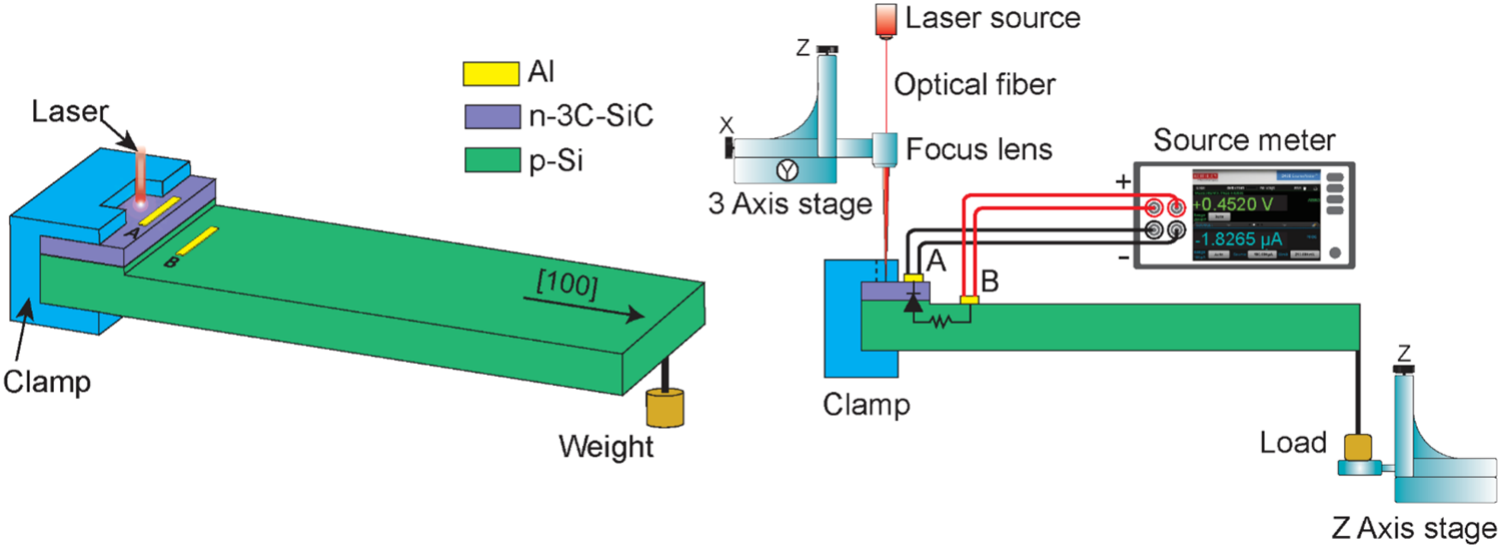
Experimental setup for characterizing the photo-piezojunction coupling effect of the n-3C-SiC/p-Si heterostructure.

Figure S2a in the Supporting Information illustrates the geometry and dimensions of the fabricated device. Figure S2b shows the transmission electron microscopy (TEM) image of a cross section of the SiC/Si heterojunction, confirming the thickness 500 nm of the SiC device. Figure S2c shows the selected area diffraction (SAED) pattern, verifying the single crystallinity of the grown 3C-SiC layer. We used the bending cantilever method to characterize the electrical properties of the n-SiC/p-Si heterostructure, where one end of the cantilever was fixed using a C-shape clamp, while weights with different values were hung on the free end, exerting tensile or compressive strain on the sensing area (Figure 2). The top side of the clamp was designed with a *U*-shape cutout, allowing illumination of laser while maintaining a consistent laser spot size and position during the strain characterization experiments. The laser system was mounted on an XYZ stage (PT3, Thorlabs) for precise control of the laser position. The laser spot size was measured using a BC100006N-VIS Beam profiler (Thorlabs) instrument; the spot diameter was 100 μm and it remained the same during all experiments. The laser power was monitored using S130C power sensors (Thorlabs) connected to a PM100D power meter (Thorlabs). The electrical characteristics were observed using a Keithley 2450 source meter. All experiments were conducted in a dark room environment at room temperature (25 °C).

## 4 Experimental Results and Discussion

**Figure 3:**
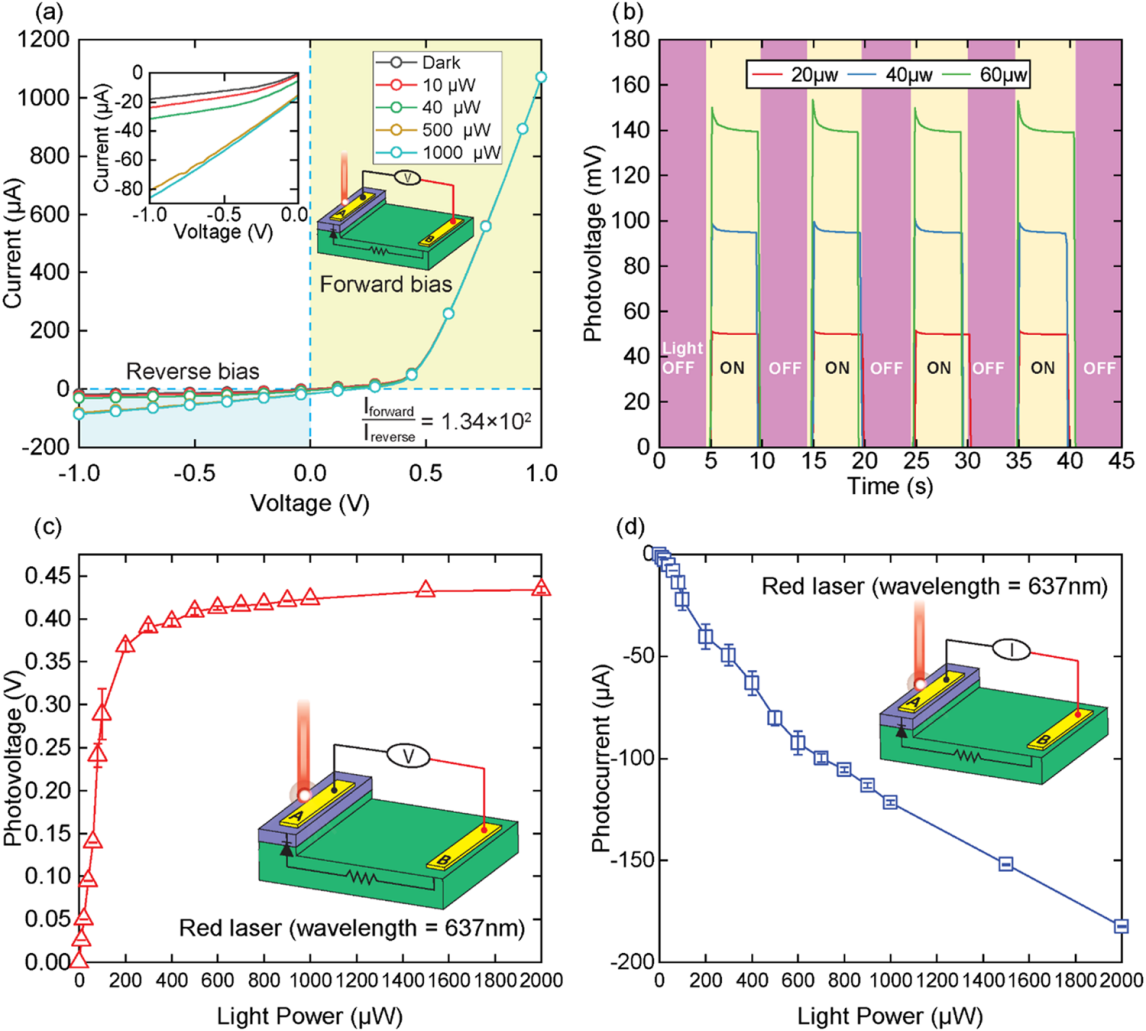
(a) Current–voltage characteristics of the 3C-SiC/Si junction exhibiting a high rectification ratio, (b) high repeatability of photovoltage generation under red laser illumination at three different powers, (c) the photovoltage, and (d) the photocurrent generation performance under red laser power from 0 to 2000 μW.

First, the current–voltage (*I*–*V*) characteristics of the device under dark condition were investigated by applying a sweeping voltage across two electrodes A and B, with the voltage value ranging from −1 to 1 V. The obtained *I*–*V* result is shown in Figure 3a. The IV curves show a highly rectifying diode property across the n-SiC/p-Si heterojunction, with the diode voltage drop of approximately 0.45 V and the diode rectification ratio value between forward current over reverse current of 1.34 × 10^2^ under dark condition. Under a nonuniform illumination of red laser, the forward current remained almost the same, while the reverse current proportionally increased with higher laser power. Compared to dark condition, the reverse current increased 4-fold to a value of −80 μA when illuminated with red laser power of 500 μW.

The photovoltaic characteristics of the n-SiC/p-Si heterojunction were investigated by nonuniformly illuminating the device under red laser wavelength 637 nm at different laser powers while monitoring the photovoltage and photocurrent generated between electrodes A and B, and the results are illustrated in Figure 3b–d. Under dark conditions, no photovoltage was generated between the two electrodes. Subsequently, when nonuniformly illuminating the sensing area with red laser, the device demonstrated excellent repeatability in photovoltage generation, achieving photovoltage values of 50, 94.8, and 139.8 mV at laser powers of 20, 40, and 60 μW, respectively (Figure 3b). This photovoltage is 1.5 times higher than the results reported in previous studies of similar 3C-SiC/Si heterostructures using the vertical photovoltage effect^28^ and 4.6 times higher than other n-3C-SiC/p-Si heterostructures utilizing the lateral photovoltage effect.^19,22^Figure 3c illustrates the photovoltage generated when the laser power increased from 0 to 2000 μW. The photovoltage generally increased with higher laser power and reached a saturation voltage of approximately 0.403 mV, at a laser power of 400 μW. Further increasing the laser power above this point only improved the photovoltage output by an insignificant amount. Figure 3d illustrates the photocurrent measured across electrodes A and B when the laser power was varied from 0 to 2000 μW. Different from the saturation in photovoltage measurement, a higher laser power resulted in a higher photocurrent, generating 182.66 μA at a laser power of 2000 μW. The photovoltage and photocurrent generations under laser illumination are due to the electron and hole concentrations increasing at electrode A and electrode B, respectively, resulting in a voltage difference *V*_AB_ when connecting the two electrodes to a voltmeter. Alternatively, if a current meter is connected between two electrodes A and B, the holes will flow from electrode B through the current meter and arrive at electrode A, resulting in a negative value on the current meter. The detailed underlying mechanisms of photovoltage and photocurrent generation will be further discussed in the following section.

**Figure 4:**
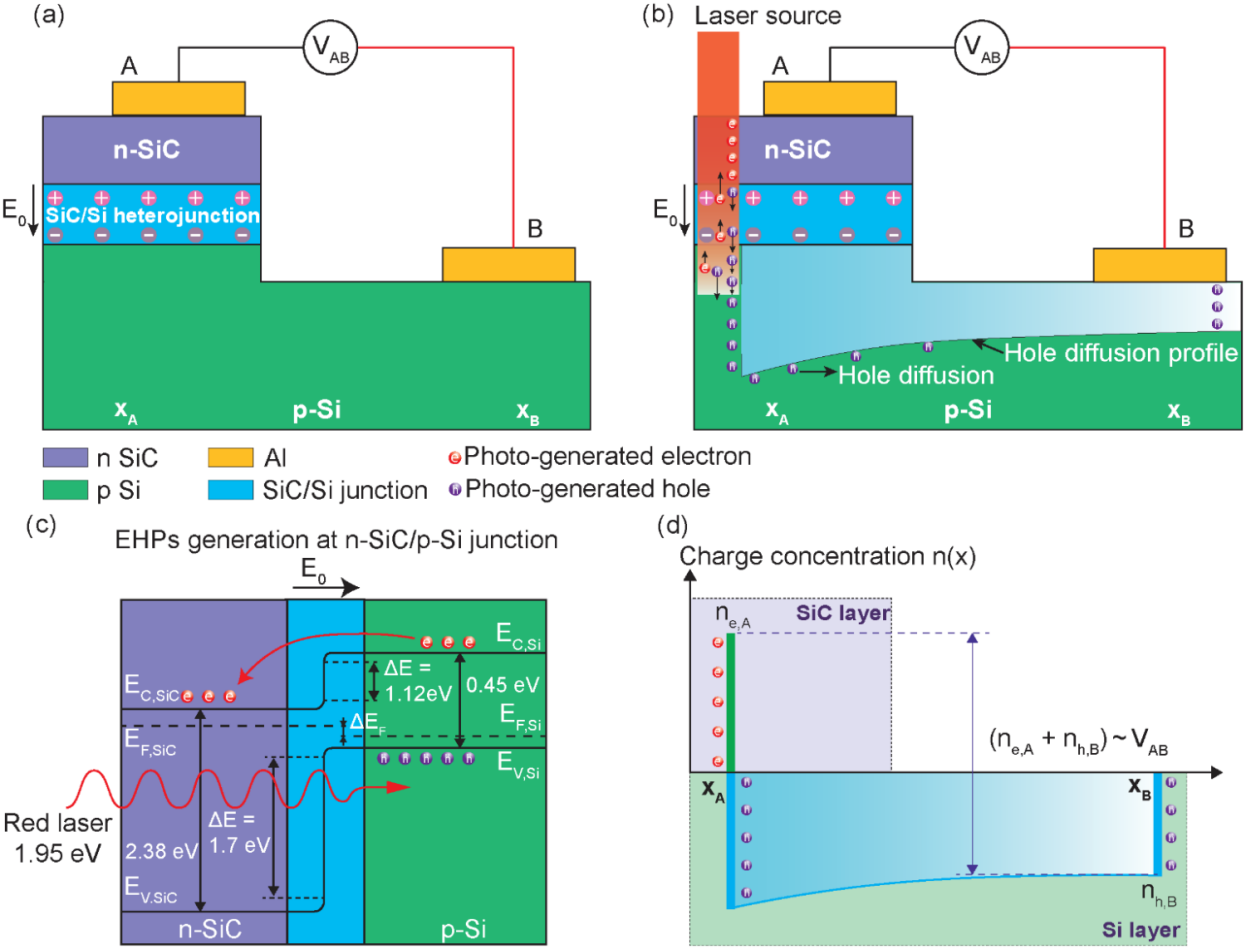
(a) Mechanism of n-3C-SiC/p-Si heterojunction forming under dark condition, and (b) underlying mechanism of photovoltage *V*_AB_ generation across electrodes A and B under red laser illumination. (c) Band diagram of the n-3C-SiC/p-Si heterojunction showing EHPs generation under laser illumination and charge carriers movement across the junction interface, leading to (d) an increase in electron and hole concentrations at electrodes A and B, respectively, and the generation of photovoltage *V*_AB_ between the two electrodes.


Based on the theory of the photovoltaic effect,^29^Figure 4 illustrates the underlying physical mechanisms of the photovoltage V_AB_ generated when red laser illuminates the n-SiC/p-Si heterojunction. Figure 4a shows the n-SiC/p-Si heterojunction in dark condition. At the interface between the n-SiC thin layer and p-Si substrate, the electrons within the n-SiC layer diffuse downward to the p-Si substrate, leaving positive charges on the SiC side, and simultaneously, holes in the p-Si substrate diffuse to the n-SiC layer, leaving negative charges on the Si side. This charge carriers diffusion eventually reaches equilibrium, creating the depletion zone, a region without charge carrier movement, and introducing an electric field *E⃗* across the depletion zone, pointing downward to the p-Si substrate. When nonuniformly illuminating the sensing area with a red laser of wavelength 637 nm, the n-SiC layer with a band-gap energy of 2.37 eV, which is larger than the photoenergy of red laser (1.95 eV), becomes transparent to the red laser photons, while the p-Si substrate with a band-gap energy of 1.12 V is suitable for absorbing red laser photons. As a result, majority of the photons in red laser pass through the SiC layer and are absorbed into the Si substrate (Figure 4b). The laser photons excite the electrons in the p-Si valence band to jump up to the conduction band, leaving holes behind in the valence band, which leads to the electron–hole pairs (EHPs) generation within the Si layer and the depletion zone under the laser spot. The built-in electric field (*E⃗*) separates the photogenerated EHPs, sweeping the electrons upwards to the n-SiC layer and holes downwards to the p-Si substrate, leading to a localized increase of electron and hole concentrations within the laser illuminated region in SiC and Si layers, respectively. Due to the generation of additional charge carriers, the Fermi energy splits into quasi-Fermi energies (*E*_F,SiC_ and *E*_F,Si_) and is given as^30,31^ (Figure 4c):

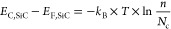
1
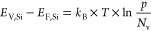
2where *k*_B_ is Boltzmann’s constant, *T* is the absolute temperature, *N*_c_ and *N*_v_ are the effective states density of the conduction band and valence band, respectively, *n* is the carrier density of electrons in n-SiC (*n* = *n*_0_ + Δ*n*, where *n*_0_ is the number of electrons in dark condition and Δ*n* is the number of additional photogenerated charge carriers). Similarly, *p* is the carrier density of holes in p-Si. Subsequently, the excess electrons diffuse upward through the SiC layer to electrode A, while simultaneously holes within the Si substrate diffuse vertically downward and then horizontally toward electrode B.

The photogenerated electrons and holes are collected at electrodes A and B, respectively, resulting in the photovoltage *V*_AB_ measured between the two electrodes (Figure 4d). The higher laser power at the sensing area generates even more electron–hole pairs, which further split the quasi-Fermi energies, resulting in a higher voltage *V*_AB_ across the electrodes, given by^31^
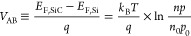
3

The photovoltaic characteristic of the n-3C-SiC/p-Si heterojunction can be qualitatively described using an equivalent electrical model, consisting of a constant current source in parallel with the junction, and is shown in Supporting Information Figure S2.^32^

The IV characteristic of a heterojunction at temperature *T* is given as^32^
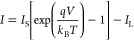
4where *I*_L_ is the photocurrent and *I*_S_ is the saturation current. The open-circuit voltage *V* = *V*_OC_ is calculated when *I = 0*, resulting in the open-circuit voltage value being defined as^32^
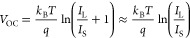
5where *q* is the charge of a single electron. It can be easily seen from eq 5 that at constant temperature the open-circuit voltage *V*_OC_ is driven by two elements, the photocurrent (*I*_L_) and the saturation current (*I*_S_). Specifically, *V*_OC_ would increase with higher *I*_L_ and/or smaller *I*_S_. However, *V*_OC_ would not increase indefinitely as the natural logarithmic term will eventually saturate when *I*_L_ reaches a sufficiently high value. This behavior agrees with the experimental result shown in Figure 3c.

Under the illumination of red laser wavelength (λ) of 637 nm, the photocurrent (*I*_L_) is defined as^32^
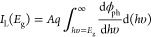
6where ϕ_ph_ is the photon flux density, *E*_g_ is the band-gap energy of the p-Si substrate, *h* is Planck constant, and υ is the photo frequency (, where *c* is the speed of light). It is clear from eq 6 that different from the saturation of the photovoltage output *V*_OC_, the photocurrent output *I*_L_ is mainly governed by the photon flux density ϕ_ph_. Specifically, considering both laser wavelength and band-gap energy of p-Si are constant, a higher laser power pushes more photons to the sensing area, resulting in a higher photon flux density and generating a higher photocurrent output, which aligns well with the experimental result shown in Figure 3d.

The saturation current (*I*_S_) is driven by the environment temperature and mobility of charge carriers μ_n_ and μ_p_, and is defined as^33^
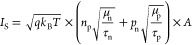
7where *A* is the effective sensing area, and τ_n_, τ_p_ are the diffusion lifetimes of electrons in p-Si and holes in n-SiC, respectively, which are related to the diffusion length as , where *D*_n_ is the diffusion coefficient given by the Einstein relation  for the electron charge carriers. Similar relations are given for the hole charge carriers.

The device’s mechanical strain-sensing capability was further investigated using the beam bending method, where a C-shape clamp was fixed at one end of the beam, while different weights were hung at the beam’s free end to exert tensile strain or pulled through a pulley system to induce compressive strain to the sensing area in the [100] direction. The weights ranging from 10 to 60 g were used, introducing strain values from 138 × 10^–6^ to 415 × 10^–6^ to the sensing area, respectively.

**Figure 5:**
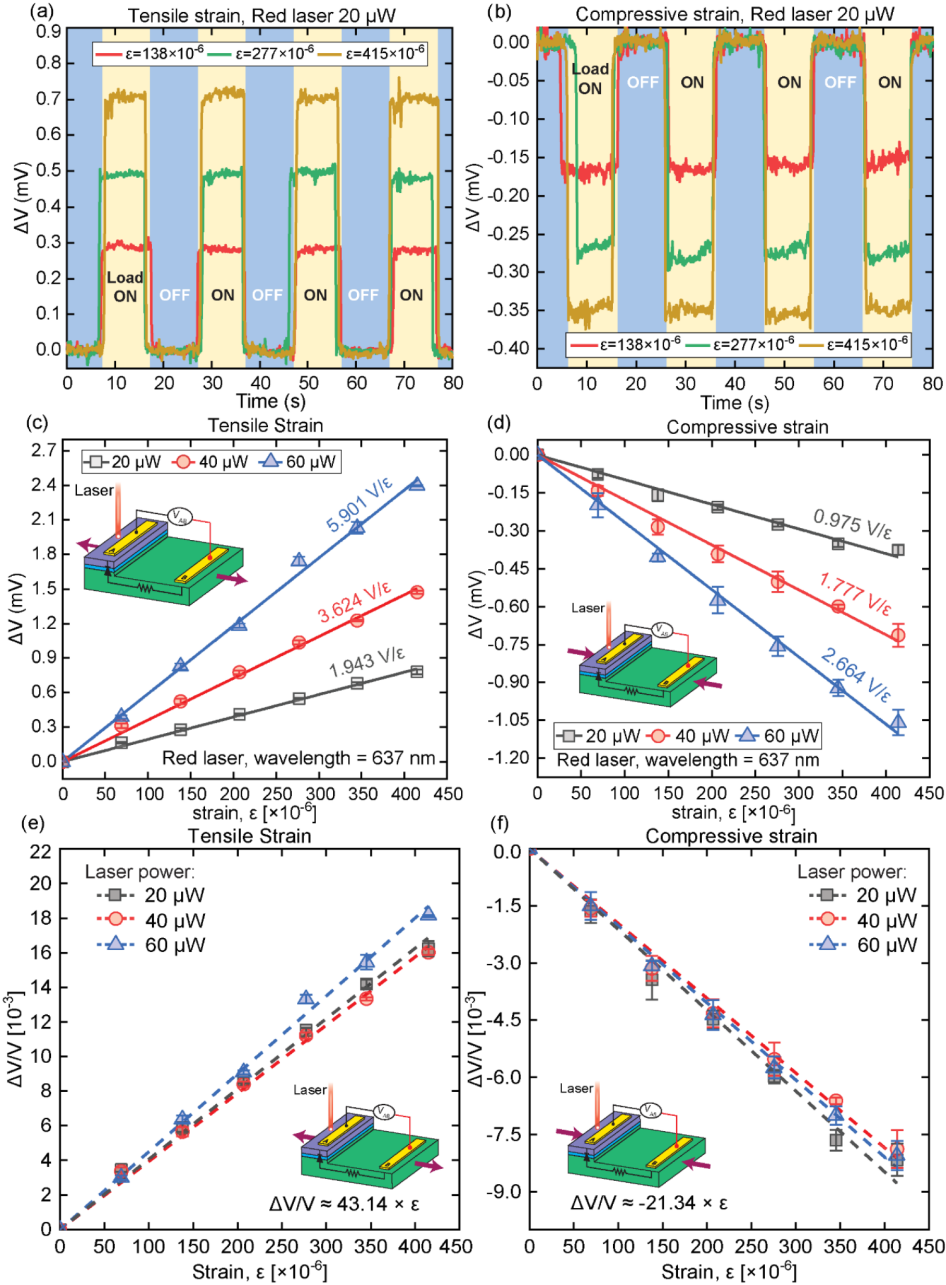
Highly reliable strain-sensing repeatability for (a) tensile strain and (b) compressive strain with consistent photovoltage output under three different strain (ε) values 138 × 10^–6^, 277 × 10^–6^, and 415 × 10^–6^ at laser power 20 μW. (c, d) Linear change of photovoltage output difference (Δ*V*) and (e, f) highly linear correlation of fractional photovoltage changes across strain (ε) values varying from 0 to 415 × 10^–6^ at three different laser powers: 20, 40, and 60 μW.

Figure 5 illustrates the strain-sensing performance via the photovoltage output difference (Δ*V*) and fractional voltage change (Δ*V*/*V*) when different strains are introduced into the device. In Figure 5a, under exposure to red laser power 20 μW, and considering the initial photovoltage output at free-strain condition is *V*_0_ (Figure 3b, *V*_0,20μW_ ≈ 50.007 mV), introducing tensile strain to the sensing area increases the photovoltage output, resulting in photovoltage output differences (Δ*V*) of 0.3, 0.5, and 0.7 mV corresponding to tensile strain (ε) values of 138 × 10^–6^, 277 × 10^–6^, and 415 × 10^–6^, respectively. The device demonstrates excellent strain-sensing repeatability, maintaining consistent Δ*V* values across all four ON-OFF tensile strain cycles. Increasing the laser power 2-fold and 4-fold raises the Δ*V* by 2-fold and 4-fold, respectively (Figure 5c). Figure 5c also shows the superior linearity relation (*R*^2^ ≈ 0.99856) between the photovoltage output difference (Δ*V*) and the applied tensile strain values ranging from 0 to 415 × 10^–6^, demonstrating strain sensitivity values of 1.943, 3.624, and 5.901 V/ε corresponding to laser powers of 20, 40, and 60 μW, respectively.

Subsequently, compressive strain was introduced to the device by using a pulley system to reverse the force direction. In contrast with the tensile strain, exerting compressive strain to the sensing area reduced the photovoltage output compared to the reference voltage *V*_0_ at free strain, resulting in the negative Δ*V* value. The photovoltage output (Δ*V*) under compressive strain is shown in Figure 5b,d.

Figure 5b clearly shows the reliable strain-sensing repeatability under laser power 20 μW, where compressive strain values of 138 × 10^–6^, 277 × 10^–6^, and 415 × 10^–6^ reduced the photovoltage by an Δ*V* amount of −0.15, −0.28, and −0.37 mV, respectively. Higher laser power further reduced the Δ*V* output. The strain sensitivities under compressive strain were 0.975, 1.777, and 2.664 V/ε corresponding to laser powers of 20, 40, and 60 μW, respectively (Figure 5d). Figure 5e,f illustrate the superior strain sensitivity with a linear relationship between the fractional voltage change (Δ*V*/*V*) and strain values from 0 to 415 × 10^–6^. It is important to note that, although the photovoltage difference (Δ*V*) increases with a higher laser power, the fractional changes (Δ*V*/*V*) were similar for all three laser powers, with the ratio between absolute fractional change (Δ*V*/*V*) and strain (ε) of approximately 43.14 and 21.34 for tensile and compressive strain, respectively, across the strain values between 0 and 415 × 10^–6^, which are approximately 4.3 times more than that of devices using a similar structure^23^ and 10 times higher than conventional metal strain gauges with a typical gauge factor of around 2.^24^

**Figure 6:**
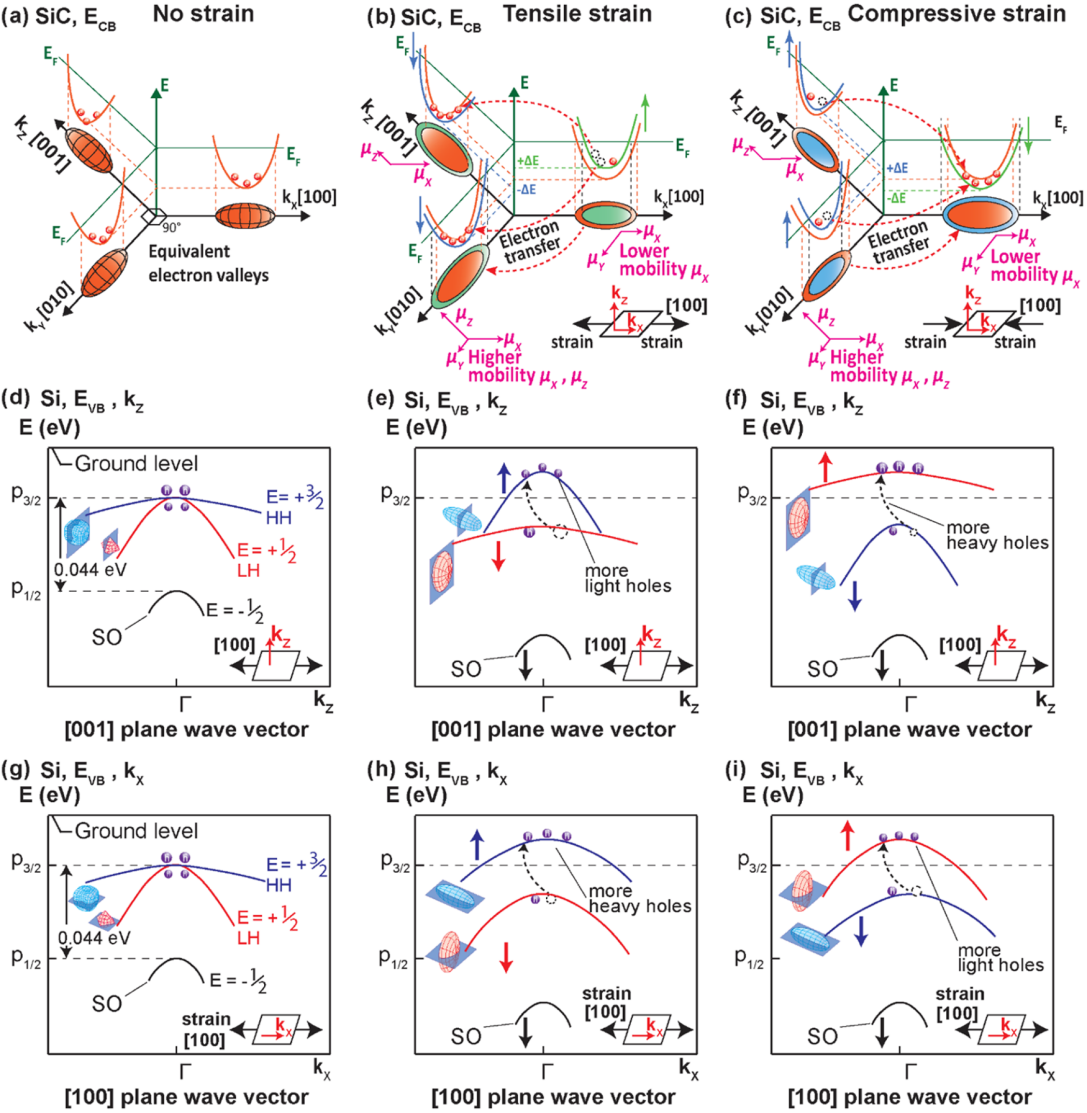
(a–c) Band shifting mechanisms of the photo-piezojunction coupling effect in the conduction band of n-SiC for strain-free condition, tensile strain, and compressive strain, respectively. (d–f, g–i) Strain-induced energy bands shifting mechanism in the valence band of p-Si for vertical *k*_Z_ direction and horizontal *k*_X_ direction, respectively.

**Figure 7:**
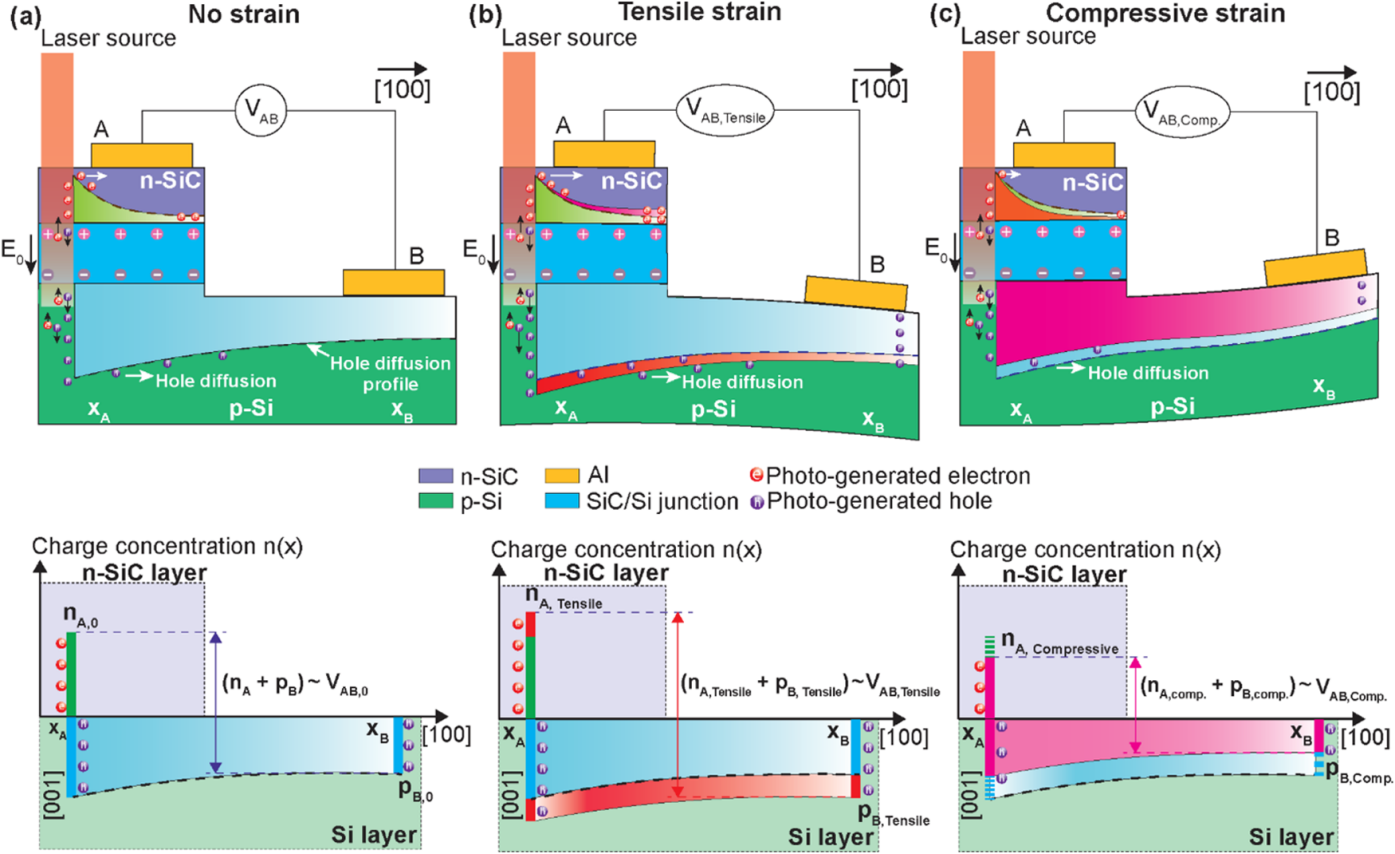
Charge carriers movement for the photo-piezojunction coupling effect in n-SiC/p-Si heterojunction under (a) strain-free condition, (b) tensile strain, and (c) compressive strain.

The underlying mechanisms for the mechanical strain coupling with the optoelectronic effect in n-3C-SiC/p-Si heterojunction can be qualitatively explained based on the theory of energy valleys warping and shifting, and charge carrier transfer in the conduction bands of n-SiC and valence bands of p-Si^34,35^ and are illustrated in Figures 6 and 7. Figure 6a–c show the strain-induced conduction band shifting mechanisms in n-SiC, Figure 6d–i show the strain-induced energy surfaces warping and valence bands shifting mechanisms in p-Si, and Figure 7 illustrates the charge carriers movement within each layer, influencing the output photovoltage *V*_AB_. The mechanisms for the photo-piezojunction effect under mechanical strain are explained as follows:

Under strain-free conditions, all conduction bands of n-SiC have uniform energy levels in all directions (Figure 6a), resulting in six energy valleys distributed equally across all directions. In the p-Si layer, the valence bands comprise a heavy hole band ( state, blue) and a light hole band (*E* =  state, red), with the energy surfaces are fluted or warped when strain is absent, due to the degeneracy at *k* = Γ (*k* = 0) for both vertical (*k*_Z_) and horizontal (*k*_X_) directions (Figure 6d,g). Therefore, in the strain-free case, the initial output voltage *V*_AB,0_ is mainly attributed to the photovoltage effect (Figure 7a).

Under tensile strain along the [100] direction, the photovoltage output *V*_AB,tensile_ increases from the initial photovoltage *V*_AB,0_ (Figure 5). Similarly, tensile strain increases the photocurrent output from the initial photocurrent, *I*_AB,0_ (Figure S4a). The physical mechanism explanations comprise the bands warping and shifting, charge carrier transfer, and changes of charge carrier mobility within the SiC/Si heterostructure, which are illustrated in Figures 6b,e,h and 7b, respectively. Figure 6b shows the n-SiC layer’s conduction bands under tensile strain, where the band edge of [100] valleys shifts upward by an amount + Δ*E*, while valley [001] and [010] band edges shift downward by −Δ*E* energy value.^35,36^ The electrons tend to transfer from the valleys with higher energy to those with lower energy, resulting in more electrons repopulating to the valleys [001] and [010]. Additionally, both valleys [001] and [010] have a higher electron mobility μ_*x*_ than valleys [100]; more electrons repopulated to these two valleys increases the total μ_*x*_ mobility in the horizontal direction (*k*_X_). For vertical direction (*k*_Z_), under tensile strain, the mobility μ_*z*_ remains approximately the same with the strain-free case as the mobility μ_*z*_ of the *k*_Y_ [010] valley is similarly high compared to the *k*_X_ [100] valley. Furthermore, the thickness of SiC is 500 nm, i.e., 200 times smaller than the laser beam diameter of 100 μm; therefore, the electron mobility in the horizontal direction μ_*x*_ is the main driving factor for the total electron mobility in the SiC layer. Consequently, under tensile strain, the SiC layer shows an increase in the horizontal mobility μ_*x*_, resulting in a larger total hole mobility and more electrons arriving at electrode A (Figure 7b).

Figure 6e,h illustrates the p-Si layer’s valence bands under tensile strain. The tensile strain along the [100] direction modifies the energy surfaces of the p-Si valence band edge by splitting the valence bands into a pair of degenerate doublets. In specific, tensile strain elongates the heavy hole band (, blue) to a prolate ellipsoid along *k*_X_ and shifts the band upward, while it squeezes the light hole band (, red) to an oblate ellipsoid and shifts the band downward.^37^Figure 6e shows the Si valence band in the vertical direction (*k*_Z_), showing that the tensile strain along [100] direction lifts the degeneracy between heavy and light hole bands, resulting in the  band (prolate ellipsoids, blue) shifting upward and the  band (oblate ellipsoids, red) shifting downward.^38^ The holes tend to transfer to a lower energy band, resulting in more holes repopulating to the  band (blue), which consequently shifts the Si valence band edge up and reduces the band gap *E*_g_ in the Si layer.

This repopulation of holes results in two changes within the p-Si substrate. First, the band gap *E*_g_ becomes smaller, allowing the Si material to absorb more laser photons and generate more EHPs within the p-Si substrate. Second, more holes are repopulated to the  band (prolate ellipsoids, blue), which has a stronger curvature along *k*_Z_ direction compared to the tensile-strained  band (oblate ellipsoids, red) and the strain-free  band (warped cubic, blue), meaning the holes transferred to this band have a higher mobility μ_*z*_ compared to the strain-free case,^39^ which contributes to a higher total hole mobility in *k*_Z_ direction and further accelerates the separation of EHPs in this direction. As a result, under tensile strain, the hole concentration increases greatly in the *k*_Z_ direction within the p-Si substrate under the laser spot (point *x*_A_, Figure 7b).

Considering the horizontal direction (*k*_X_) in p-Si substrate, tensile strain in the [100] direction also lifts the degeneracy between heavy and light holes, with holes repopulating to the  band (blue) (Figure 6h); however, different from the *k*_Z_ direction, the tensile-strained  band (prolate ellipsoids, blue) in *k*_X_ direction has a curvature slightly stronger than the strain-free  band (warped cubic, blue), and the tensile-strained  band (oblate ellipsoids, red) has a curvature slightly weaker than the strain-free  band (warped octahedron, red), meaning the holes repopulated due to tensile strain have a mobility slightly higher than that of strain-free condition and increase the total hole mobility in horizontal direction. However, the effect of strain in horizontal direction is insignificant as the tensile strain was intentionally designed along the [100] direction, i.e., the longitudinal axis of p-Si, which has a minimal piezoresistive effect for p-Si.^27^ This results in an almost identical horizontal hole mobility between the strain-applied and strain-free condition, allowing the holes to diffuse horizontally in the p-Si substrate along the [100] direction with the same diffusion curve, compared to that of the strain-free case, to electrode B (point *x*_B_, Figure 7b) and ultimately allowing the investigation to focus only on the strain effect for the vertical direction i.e., [001] direction in n-3C-SiC/p-Si heterojunction.

Figure 7b illustrates the effect of tensile strain on the charge carriers movement within the n-3C-SiC/p-Si heterojunction device under the illumination of a red laser. The tensile strain along the [100] direction enhances the electron mobility μ_*x*,electron_ in the SiC layer, allowing more electrons to arrive at electrode A, and simultaneously improves the EHP generation in the Si layer through the Si band-gap reduction. These additional EHPs are further separated more effectively due to the boosting of hole mobility μ_*z*,hole_ in vertical direction (*k*_Z_) during tensile strain, allowing more holes to diffuse toward electrode B. As a result, a larger photovoltage *V*_AB_,_tensile_ is outputted as more electrons and holes can be collected at electrodes A and B, respectively.

In reverse, compressive strain along the [100] direction reduces the photovoltage output V_AB_,_compressive_ from the initial strain-free photovoltage *V*_AB,0_ (Figure 5) and similarly reduces the photocurrent output from the initial strain-free photocurrent *I*_AB,0_ (Figure S4b). The underlying energy band shifting mechanism and charge carriers transfer in n-3C-SiC/p-Si heterojunction under compressive strain are illustrated in Figures 6c,f,i, and 7c, respectively.

In the n-SiC layer, compressive strain shifts the valleys [001], [010] upward and valley [100] downward, resulting in more electrons repopulating to valley [100], which has a lower mobility μ_*x*_, meaning less electrons can reach electrode A in the SiC layer. For the Si layer, similar to the tensile strain case, the compressive strain also elongates the , band (blue) to a prolate ellipsoid along *k*_X_, and squeezing light hole band (*E* = , red) to an oblate ellipsoid;^37^ however, contrary to the tensile strain, the  band (prolate ellipsoid, blue) shifts downward, while the  band (oblate ellipsoid, red) shifts upward.^38^ As a result, more holes are repopulated to the  band (red), with a lower vertical hole mobility μ_*z*,hole_, resulting in a less effective EHPs separation, which reduces the hole concentration in the Si layer under the laser spot (point *x*_A_, Figure 7c), and consequently less holes can reach electrode B (point *x*_B_, Figure 7c). Therefore, under compressive strain, a smaller photovoltage *V*_AB,compressive_ is generated as fewer electrons and holes reach electrodes A and B, respectively. It is worth noting that, although the EHPs separation is less effective under compressive strain, the lifting of the  band (oblate ellipsoid, red) reduces the p-Si band-gap energy *E*_g_, and contributes to more EHPs generation, which partially attenuates the reduction of photovoltage *V*_AB,compressive_ when compressive strain is applied. This hypothesis aligns well with the experimental results, showing a larger (Δ*V*/*V*)/ε ratio for the tensile strain compared to that of the compressive strain (Figure 5e,f).

## 5 Conclusions

In conclusion, we have investigated the photo-piezojunction coupling effect in the n-3C-SiC/p-Si heterojunction with applications for mechanical strain sensors capable of harvesting photon energy. A proof-of-concept cantilever device has been fabricated, incorporating a simple yet effective and reliable heterostructure design, allowing efficient photon energy harvesting while consistently sensing the external mechanical strain applied. The device demonstrates superior light-harvesting capability, outputting a photovoltage of 25.54 mV under the illumination of red laser with a laser power as small as 10 μW. This photovoltage is 1.5 times and 4.6 times higher than those of previously reported devices relying on vertical and lateral photovoltaic design, respectively. Additionally, the device exhibits excellent strain-sensing capability across the strain values ranging from 0 to 415 × 10^–6^, achieving a highly linear Δ*V*/*V* with a strain sensitivity ratio (Δ*V*/*V*)/ε of 43.14 and 21.34 for tensile and compressive strain, respectively, i.e., 4.3 times larger than that of devices reported in the previous studies utilizing a similar strain-sensing technique, and 10 times more than that of conventional metal strain gauges, which has a typical ratio of only approximately 2.

The proposed device also demonstrates excellent reliability, capable of generating consistent photovoltage values at different illuminating laser powers and accurately monitoring the external strains applied during multiple on–off cycles of laser illumination and strain application. The underlying physical mechanisms of the photovoltage generation in the strain-free condition have been explained based on the theory of the photovoltaic effect and qualitatively described using an equivalent electrical model. Furthermore, the mechanisms for mechanical strain sensing have been explained based on the theory of energy valleys warping and shifting, and charge carrier transfer in both the conduction bands of n-3C-SiC and the valence bands of p-Si, which agrees well with the experimental results.

Our future work involves developing a comprehensive theoretical model to quantitatively characterize the photo-piezojunction coupling effect, which will significantly benefit the development of future sensor systems. The findings in this study substantially advance the understanding of the photo-piezojunction coupling effect in n-3C-SiC/p-Si heterostructures, providing a foundation for the design of better multifunctional sensor systems capable of harvesting photon energy.

## Supporting Information Available

Fabrication process of the device (Figure S1); geometry of the fabricated device (Figure S2); idealized equivalent circuit (Figure S3); photocurrent response under strain (Figure S4) (PDF)
