# Supplementary material for: Photo-Piezojunction Coupling Effect in n-3C-SiC/p-Si Heterojunction – A Platform for Self-Powered Strain-Sensing Applications
Source: ACS Appl Mater Interfaces. 2025 Apr 17;17(17):25489–99. doi: 10.1021/acsami.5c02290 (PMC12051830; doi:10.1021/acsami.5c02290)
Supplement: Supplementary file 1 — am5c02290_si_001.pdf [file am5c02290_si_001.pdf]

Supporting Information

Photo-Piezojunction Coupling Effect in n-3C-SiC/p-Si Heterojunction – A Platform for Self-Powered Strain Sensing Applications

D.H. Dang Tran [a]*, Tuan-Hung Nguyen[a], Cong Thanh Nguyen[a], Erik W. Streed[b], Nam-Trung Nguyen[a], Van Thanh Dau[c]*, and Dzung Viet Dao [a,c]*

[a]*Queensland Micro- and Nanotechnology Centre, Griffith University, 170 Kessels Road, Brisbane, Queensland, 4111, Australia.*

[b]*Institute for Glycomics and Centre for Quantum Dynamics, Griffith University, Parklands Drive, Gold Coast, Queensland, 4222, Australia.*

[c]*School of Engineering and Built Environment, Griffith University, Parklands Drive, Gold Coast, Queensland, 4222, Australia.*

**Email: dang.tran@griffithuni.edu.au*

1. Sample Fabrication

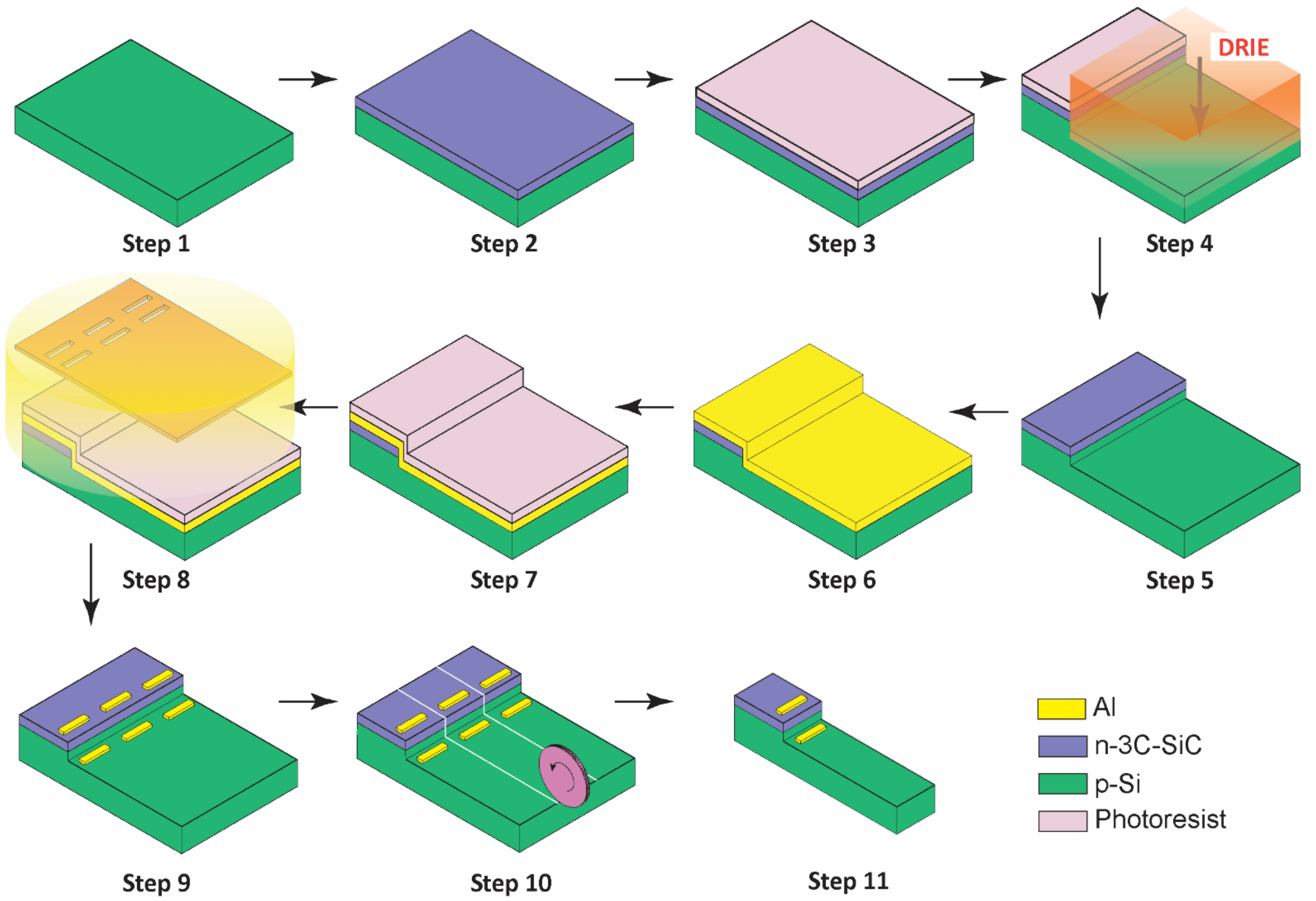

Figure S1: Fabrication process of the proof-of-concept device for characterizing the photo-piezojunction effect in n-3C-SiC/p-Si heterojunction.

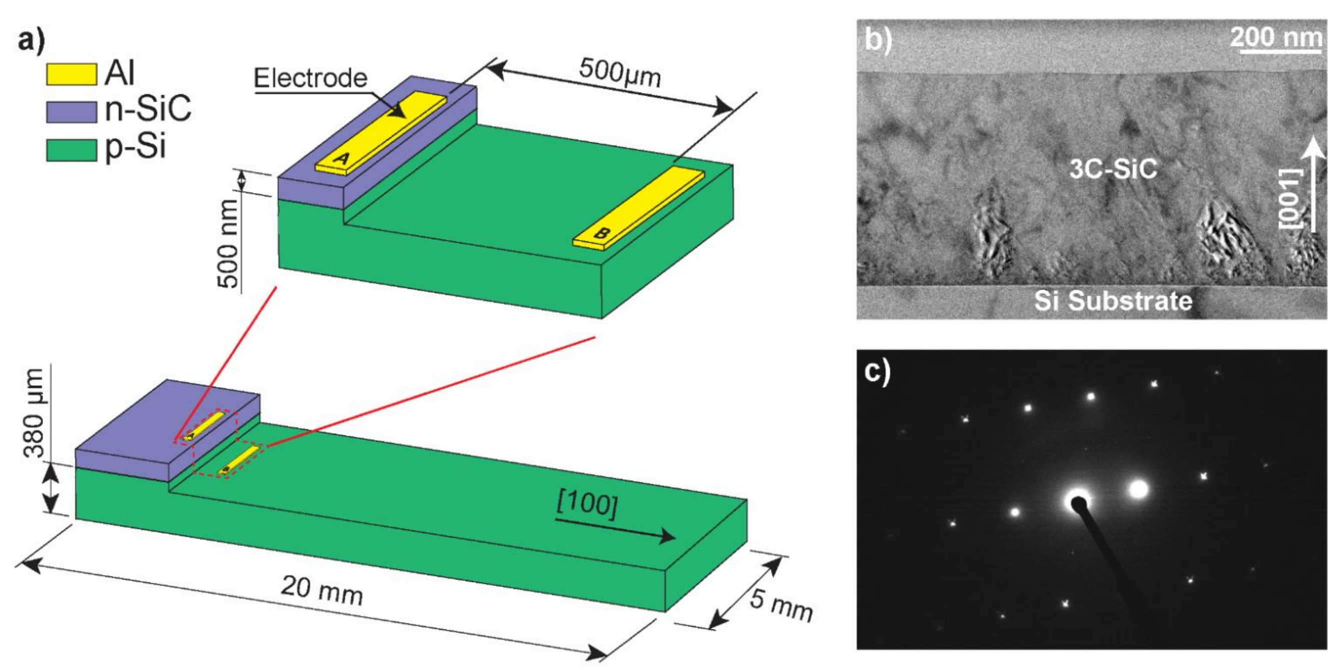

Figure S2: (a) Geometry of the fabricated self-power strain sensing device, (b) SEM image of the fabricated sample and (c) TEM image of a cross-section of the n-3C-SiC/p-Si heterojunction.

2. Idealized equivalent circuit

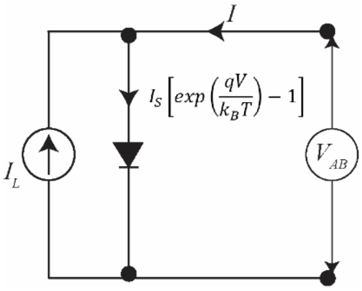

Figure S3: Idealized equivalent circuit of n-3C-SiC/p-Si diode device.

3. Photocurrent response under tensile and compressive strain

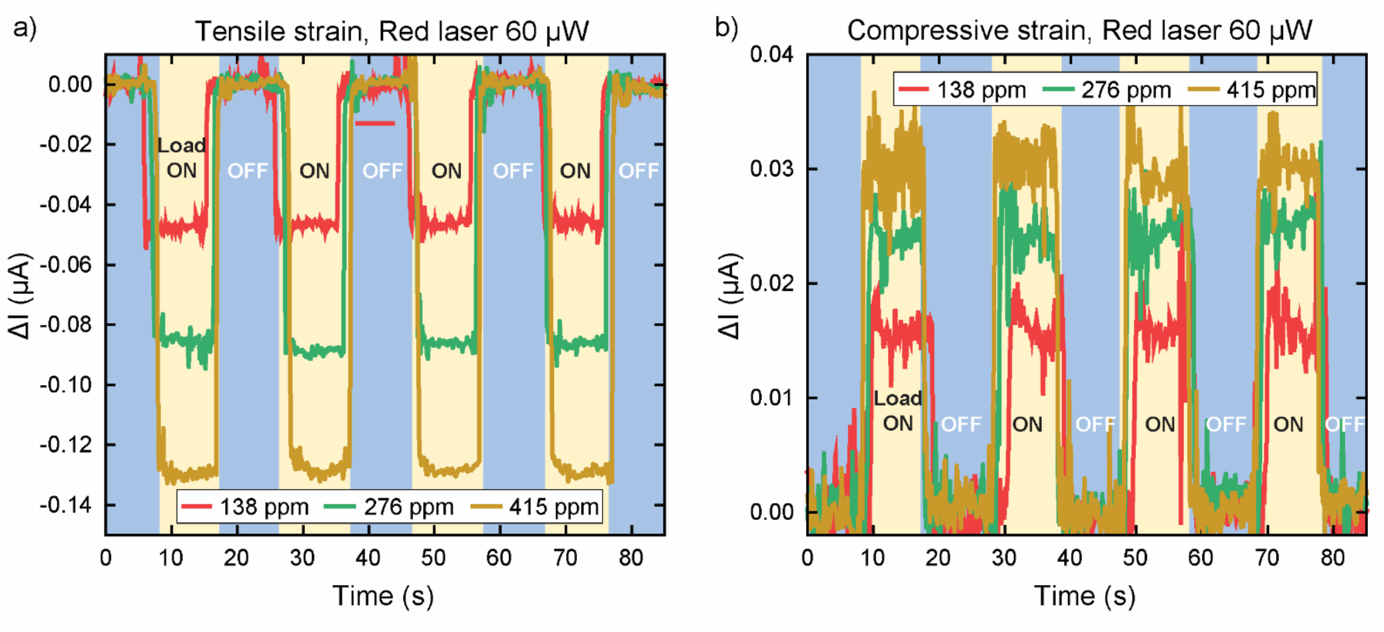

Figure S4: Highly reliable strain sensing repeatability with consistent photocurrent output under three different strain (ε) values 138×10^{-6}, 277×10^{-6} and 415×10^{-6} at laser powers of 60 µW. (a) under tensile strain, the photocurrent further increased by a negative ΔI value from the initial strain-free photocurrent I_0 ($I_{0,\ 60\ \mu W}$ = -7.892 µA), while reversely, (b) compressive strain reduced the initial strain-free photocurrent I_0 by a ΔI value.
